# A New Chloroplast DNA Extraction Protocol Significantly Improves the Chloroplast Genome Sequence Quality of Foxtail Millet (*Setaria italica* (L.) P. Beauv.)

**DOI:** 10.1038/s41598-019-52786-2

**Published:** 2019-11-07

**Authors:** Dan Liu, Yanjiao Cui, Suying Li, Guihua Bai, Qiang Li, Zilong Zhao, Dan Liang, Conglei Wang, Jianhe Wang, Xiaowei Shi, Chao Chen, Gang Feng, Zhengli Liu

**Affiliations:** 1 Department of Life Sciences, Tangshan Normal University, Tangshan, 063000 China; 2 Tianjin Crop Research Institute, Tianjin Academy of Agricultural Sciences, Tianjin, 300384 China; 3 United States Department of Agriculture-Agricultural Research Service, Hard Winter Wheat Genetics Research Unit, 4008 Throckmorton Hall, Manhattan, KS 66506 USA

**Keywords:** Biological techniques, Plant sciences

## Abstract

The complexity of the leaf constitution of foxtail millet (*Setaria italica* (L.) P. Beauv.) makes it difficult to obtain high-purity cpDNA. Here, we developed a protocol to isolate high-quality cpDNA from foxtail millet and other crops. The new protocol replaces previous tissue grinding and homogenization by enzyme digestion of tiny leaf strips to separate protoplasts from leaf tissue and protects chloroplasts from damage by undue grinding and homogenization and from contamination of cell debris and nuclear DNA. Using the new protocol, we successfully isolated high-quality cpDNAs for whole-genome sequencing from four foxtail millet cultivars, and comparative analysis revealed that they were approximately 27‰ longer than their reference genome. In addition, six cpDNAs of four other species with narrow and thin leaf blades, including wheat (*Triticum aestivum* L.), maize (*Zea may* L.), rice (*Oryza sativa* L.) and sorghum (*Sorghum bicolor* (L.) Moench), were also isolated by our new protocol, and they all exhibited high sequence identities to their corresponding reference genomes. A maximum-likelihood tree based on the chloroplast genomes we sequenced here was constructed, and the result was in agreement with previous reports, confirming that these cpDNA sequences were available for well-supported phylogenetic analysis and could provide valuable resources for future research.

## Introduction

Chloroplasts serve as important cytoplasmic organelles in higher plants. Multiple biochemical processes take place in chloroplasts, including photosynthesis, nitrogen metabolism, sulfate reduction, and synthesis of starch, amino acid and lipid. As semi-autonomous cellular organelles, chloroplasts possess their own independent genome and a full complement of transcription and translation machinery to express their genetic information^1^. The chloroplast DNA (cpDNA) contains 110–130 genes with sizes varying from 120–160 kilobases (kb) in most plants^1–3^, and only a part of the cpDNA is circular^4^. Most of these genes code for proteins involved in photosynthesis or gene expression, with the remainder as transfer RNA (tRNA) or ribosomal RNA (rRNA) genes^2^.

In green plants, the chloroplast genome is much simpler in structural complexity than the nuclear genome and generally has a highly conserved organization and gene content across species^5^. Most chloroplast genomes have a quadripartite structure that includes two copies of an inverted repeat (IR) that separates the genome into large and small copy regions (LSC and SSC)^6,7^. In addition, cpDNA is usually maternally inherited without genetic recombination^8,9^. Thus, the chloroplast genome sequence can provide a wealth of information for researchers on plant phylogeny, molecular ecology, comparative genomics, population genetics and evolution and also contribute to chloroplast transformation for crop improvement. Sequencing of high-quality cpDNA is the prerequisite for such studies, and cpDNA fragments amplified from total genomic DNA by polymerase chain reaction (PCR) are currently used for chloroplast genome sequencing. However, this sequencing-quality DNA preparation method is known to be time consuming and difficult to implement when gene organization differs among different plant species^10,11^ and the existence of promiscuous DNA sequences transferred from chloroplasts and mitochondria to the nucleus also affects the reliability of the results from related studies^12–14^. Thus, a protocol that attempts to separate chloroplasts directly before cpDNA isolation will improve the quality of cpDNA for sequencing.

Currently, cpDNA isolation from fresh plant materials uses high-salt buffers^15^, a sucrose density gradient or Percoll gradient to separate chloroplast first^16^, and then DNase to remove nuclear DNA^17^. All methods need to grind and homogenize leaf tissues and separate and purify chloroplasts by gradual centrifugation. However, grinding and homogenization usually destroy a portion of the chloroplasts, and cell debris and nuclear DNA can easily contaminate isolated cpDNA. Therefore, extraction of high-quality cpDNA is extremely difficult in some species, such as foxtail millet (*Setaria italica* (L.) P. Beauv), rice (*Oryza sativa* L.) and other Poaceae crops. Their leaf blades are relatively thin and fibrous with low chloroplast content in mesophyll cells, and the abundant wax, cuticle and silica on the leaf surface also make it hard to break cells to release chloroplasts^18^. Thus, solving these technical issues will significantly improve the quality of cpDNA isolation from foxtail millet.

In this study, we report a new protocol for the efficient isolation of high-quality cpDNA from foxtail millet. The new protocol significantly increases chloroplast and cpDNA purity and eliminates nuclear and mitochondria DNA contamination. Using this protocol, intact cpDNAs with sequencing quality were isolated from four cultivars of foxtail millet and six cultivars of wheat (*Triticum aestivum* L.), maize (*Zea may* L.), rice and sorghum (*Sorghum bicolor* (L.) Moench). The availability of these chloroplast genomes was proven by comparative analysis and phylogenetic analysis, and they will provide valuable information for future research.

## Results

### Isolation of chloroplast DNA

**Figure 1:**
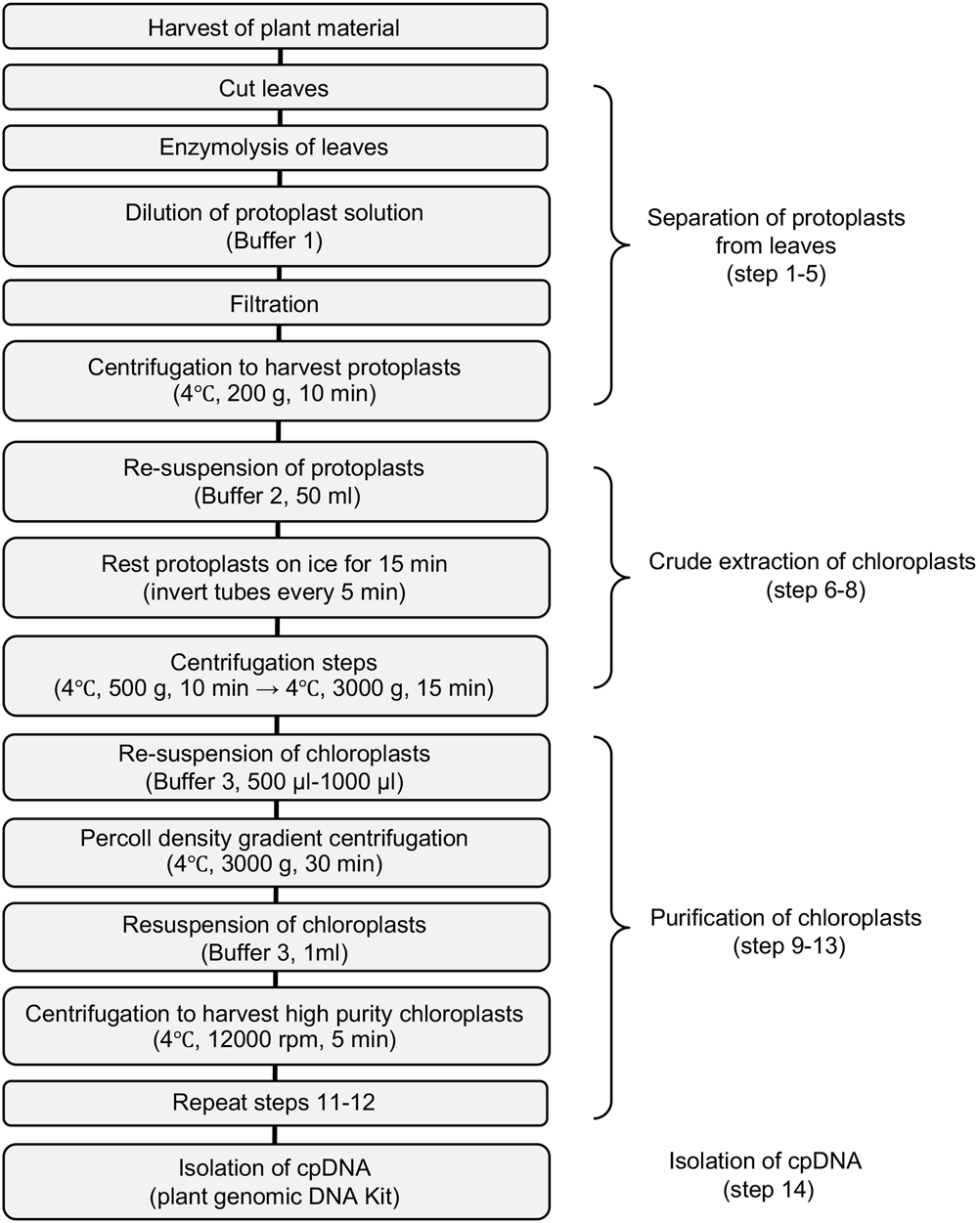
Flowchart showing the major steps for the isolation of high-purity chloroplast DNA from foxtail millet using the new optimized protocol.

To isolate cpDNA in foxtail millet, the first step is to separate chloroplasts from other components. In previous protocols, this step was achieved by grinding and homogenizing leaf samples in an isolation buffer. However, abundant wax, cuticle and silica deposited on the leaf surface make it difficult to break cells and release sufficient chloroplasts into isolation buffer. The new protocol uses several novel strategies to obtain pure chloroplasts from cells (Fig. 1). First, the tissue grinding procedure was replaced by cutting leaves into tiny pieces using a surgical blade, which minimized the contamination of cell debris and chloroplast damage. In addition, mechanical wounding from cutting can increase contact between enzymes and cells. Thus, this new method protects chloroplast intactness and facilitates the release of chloroplasts into extraction buffer. Then, cellulase and macerozyme were used to effectively separate large quantities of intact protoplast from leaf material after centrifugation at 200 g for 10 min. Eliminating the grinding and homogenizing step also minimized the contamination of cell debris and nuclear DNA from grinding tissue. Second, the protoplasts were re-suspended in Buffer 2 followed by two additional centrifugation steps at 500 g for 10 min and 3000 g for 15 min to separate chloroplasts from cell debris and mitochondrial DNA and collect crude chloroplast precipitate based on the difference in sedimentation rates between mitochondria and chloroplast. Third, an extra Percoll density gradient centrifugation step was used to purify isolated chloroplasts. The crude chloroplast pellet was dissolved in Buffer 3 and layered onto a Percoll gradient (10–50% for foxtail millet). After centrifugation at 3000 g for 30 min, intact and pure chloroplasts were recovered from the Percoll interface, and additional nuclear DNA was eliminated.

The new method was used to extract cpDNAs from ten genotypes of different species, including four foxtail millet cultivars (Gu56A, Gu572A, Datong28 and Datong29lv), three wheat cultivars (Jinqiang8, Jinnong6 and Lunxuan987), one maize hybrid cultivar (Zhengdan958), one Japonica rice cultivar (Jingeng818) and one sorghum cultivar (SbJ200), revealing that this protocol was quite valuable for the isolation of cpDNAs from not only foxtail millet but also other Poaceae species with narrow and thin leaves.

### Chloroplast genome sequencing

To evaluate the quality of the cpDNAs isolated using the new protocol, we sequenced the complete chloroplast genomes of the five Poaceae crops mentioned above using Illumina next-generation sequencing technology. cpDNAs isolated using the new protocol generated a higher proportion (4.22–12.35%) of mapped reads on the reference chloroplast genomes than those (1% to 4%) from cpDNAs isolated using old protocols^19^, confirming that the new protocol significantly improved the purity of cpDNAs and generated enough high-quality cpDNAs to assemble the entire chloroplast genome; thus, this protocol has solved previous issues associated with the isolation of cpDNAs from plant species with narrow and thin leaves.

**Figure 2:**
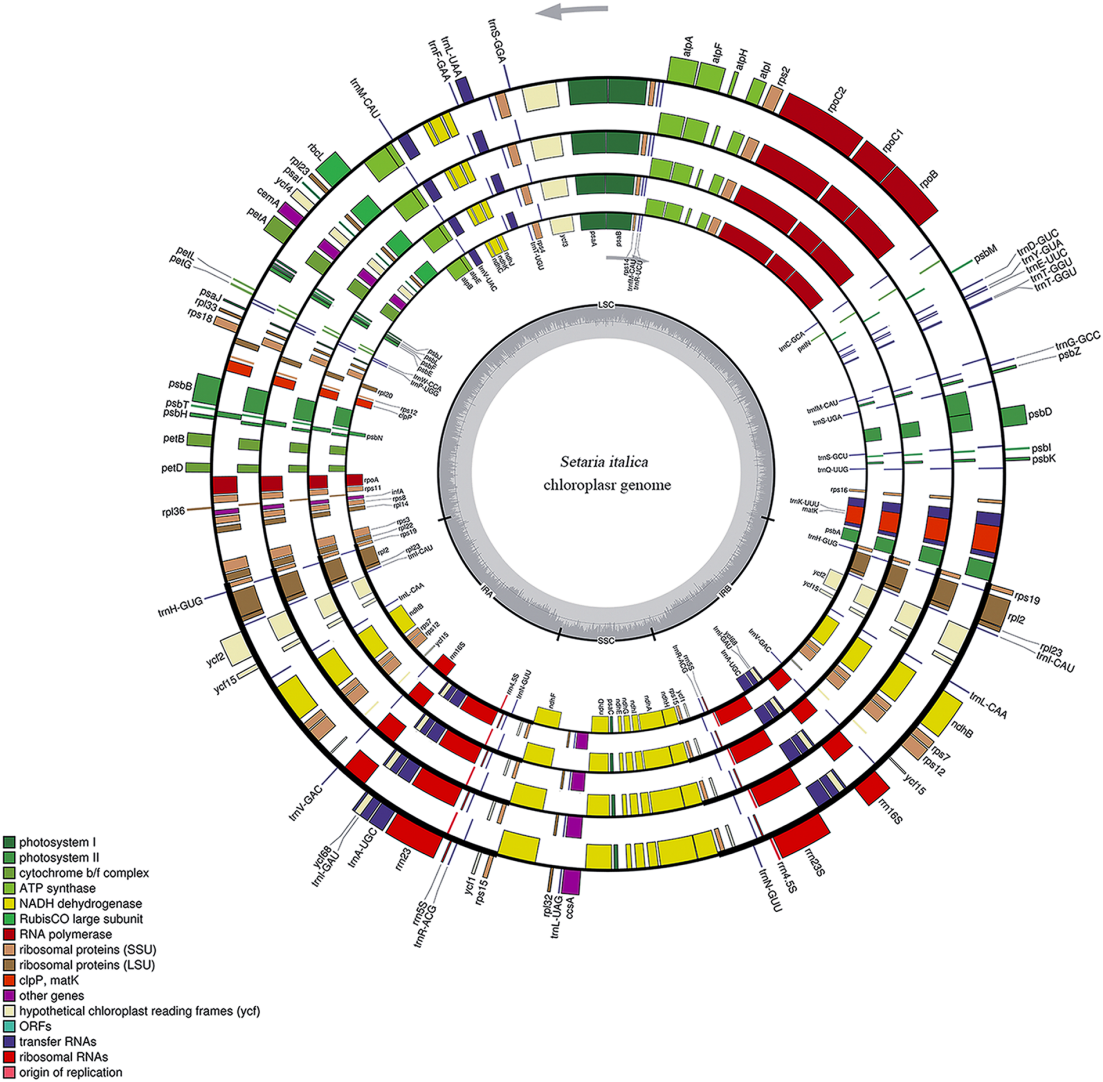
Genome structure and mapping of genes in the foxtail millet chloroplast genome. The foxtail millet cultivars from inside to outside are Gu56A, Gu572A, Datong28 and Datong29lv, respectively. The thick lines indicate the extent of the IRA and IRB, which separate the genome into the SSC and LSC regions. Genes on the outside of the map are transcribed in the counterclockwise direction, and genes on the inside of the map are transcribed in the clockwise direction. Genes are colored according to their functions as shown in the legend.

The results showed that all chloroplast genomes we sequenced had a typical quadripartite structure, including two IR regions, an LSC region and an SSC region (Fig. 2, Supplementary Figs S1–S4), with conservative genome size and gene content (Table 1, Supplementary Tables S1–S2). The sequenced chloroplast genomes are 140,454–140,659 bp long in the typical C_4_ plants maize and sorghum and 134,435–136,482 bp long in the C_3_ plants wheat and rice. The chloroplast genome of foxtail millet is 139,159–139,261 bp in length, including 129–131 genes with 90–92 genes encoding amino acids, and showed 45 Single Nucleotide Polymorphisms (SNPs) and nine Insertions and Deletions (InDels) among different cultivars, which can be used for marker development for breeding, population genetics and evolution studies.

**Table 1:** Statistics on the basic features of the chloroplast genomes of four foxtail millet cultivars sequenced in our study.

	Gu56A	Gu572A	Datong28	Datong29lv
Length (bp)	139159	139261	139218	139176
GC content (%)	39.65	38.66	38.64	28.64
AT content (%)	61.35	61.34	61.36	61.36
LSC length (bp)	81883	81985	81955	81902
SSC length (bp)	12530	12530	12529	12528
IR length (bp)	22373	22373	22367	22373
Gene number	130	131	129	130
Gene number in IR regions	37	37	37	37
Protein-coding gene number	91	92	90	91
Protein-coding gene (%)	70	70.23	69.77	70
rRNA gene number	7	7	7	7
rRNA (%)	5.38	5.34	5.43	5.38
tRNA gene number	32	32	32	32
tRNA (%)	24.62	24.43	24.81	24.62

### Comparative analysis of chloroplast genomes

To further examine the quality of cpDNAs isolated using the new protocol, we aligned the ten sequenced chloroplast genomes with their corresponding reference genome sequences in National Center for Biotechnology Information (NCBI, https://www.ncbi.nlm.nih.gov/) using the web-based VISualization Tool for Alignments (mVISTA, http://genome.lbl.gov/vista/index.shtml)^20^. The chloroplast genome sequence of the maize hybrid Zhengdan958 is identical to its reference chloroplast genome (cultivar B73, AY928077.1) (Supplementary Fig. S5). Rice cultivar Jingeng818 and sorghum cultivar SbJ200 exhibited high sequence identity to their reference chloroplast genomes (Nipponbare, GU592207.1 for rice and BTx623, EF115542.1 for sorghum) (Supplementary Figs S6–S7). Those results showed that the new protocol produced high-quality chloroplast cpDNAs for sequencing, and the derived genome sequences should be reliable for further studies.

The sequenced chloroplast genomes of wheat varied with variety. The chloroplast genome sequence of Jinnong6 was 647 bp (4.76‰) longer, and those of Jinqiang8 and Lunxuan987 were 261 bp (1.92‰) and 398 bp (2.93‰) shorter, respectively, than the Chinese Spring chloroplast reference genome (KJ614396.1, 135,835 bp). Several large InDels in intergenic spacers (IGS) and genic regions of *psbA*, *rps7* and *rrn23S* accounted for the majority of the size variation (Supplementary Fig. S8).

**Figure 3:**
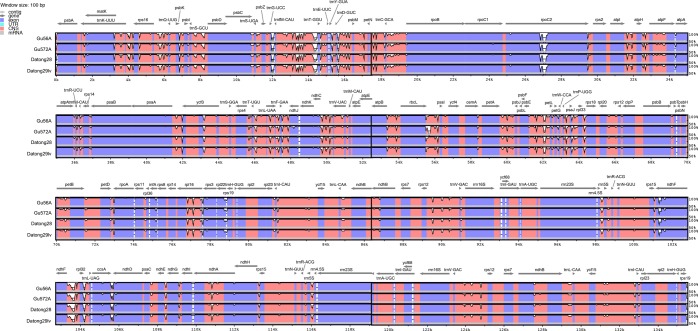
Alignment and percentage identity of complete chloroplast genome sequences of five foxtail millet cultivars using mVISTA. Measures of similarity are relative to the reference chloroplast genome NC_022850.1. Exons are shaded in dark blue, and the conserved non-coding sequences (CNS) are shaded in pink. Arrows indicate positions of annotated genes in reference sequences.

The complete chloroplast genome sizes of the sequenced four foxtail millet cultivars were 3,643–3,745 bp longer than that of their reference genome (NC_022850.1, 135,516 bp in length). The mVISTA alignment results showed that a total of 337 sequence variations (88 insertions and 249 deletions) were detected in NC_022850.1 when compared with the four foxtail millet chloroplast genomes sequenced in our study (Fig. 3, Supplementary Table S3). The majority (272) of InDels were located in IGS of the chloroplast genome, and a small fraction of InDels were distributed in exons (22) or introns (43) of different genes. Among the 337 variations, 219 (64.99%) were 1-10-bp-long small InDels, 89 (26.41%) were 11–40 bp in length, and the remaining 29 (8.60%) were 41–200 bp long. We also found that the chloroplast genome size difference between the sequenced four foxtail millet cultivars and NC_022850.1 foxtail millet reference (27‰) was similar to that between foxtail millet and wheat (19.6–28.2‰), slightly smaller than that between foxtail millet and rice (35.14–35.9‰), and significantly larger than those between foxtail millet and maize (8.6–9.3‰) or sorghum (10.0–10.8‰) (Table 2), suggesting that the size differences between the four foxtail millet chloroplast genomes sequenced here and NC_022850.1 reached or even exceeded the degree of differences between chloroplast genomes of some different species.

**Table 2:** Size differences (‰) between four chloroplast genomes of foxtail millet cultivars and six chloroplast genomes of other grasses (wheat, maize, rice and sorghum) sequenced in our study.

	56A	572A	Datong28	Datong29lv
56A				
572A	0.73			
Datong28	0.42	0.30		
Datong29lv	0.12	0.61	0.30	
Jinnong6	19.61	20.36	20.05	19.74
Jinqiang8	26.44	27.20	26.88	26.57
Lunxuan987	27.48	28.23	27.92	27.61
Zhengdan958	9.30	8.57	8.88	9.18
Jingeng818	35.14	35.90	35.58	35.27
SbJ200	10.78	10.04	10.35	10.66

### Phylogenetic analysis

**Figure 4:**
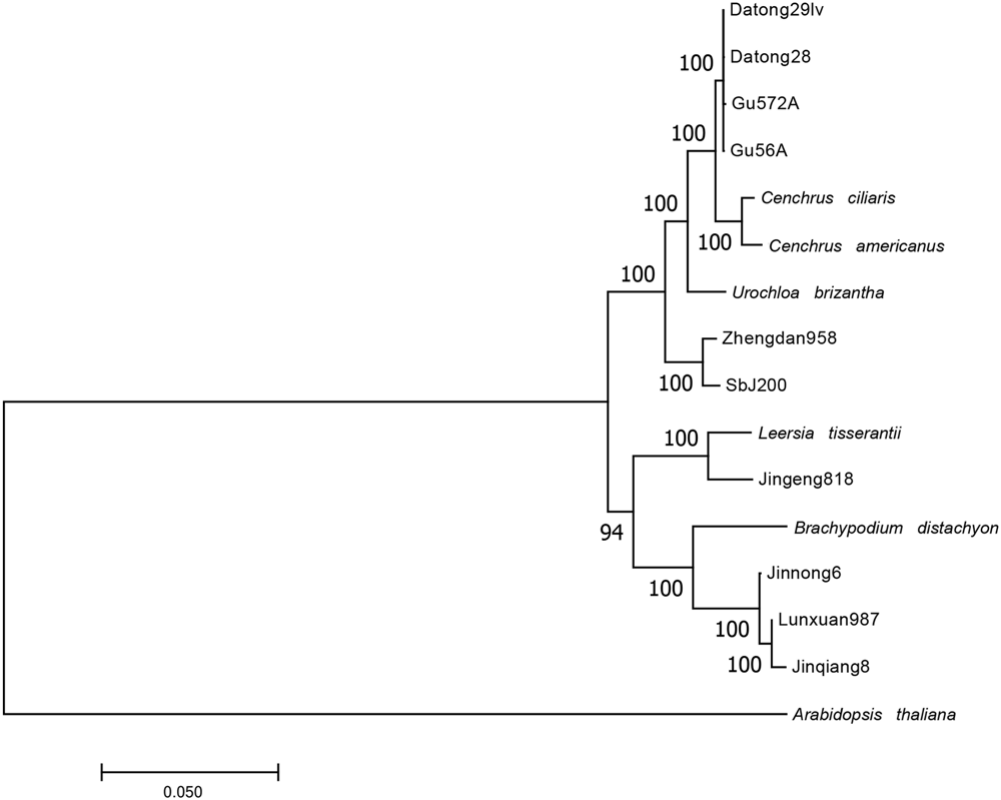
The maximum-likelihood tree of fifteen species of Poaceae based on complete chloroplast sequences. Bootstrap support values are shown on the nodes. The chloroplast genome of *Arabidopsis thaliana* was used as an out-group.

To assess their phylogenetic relationships, a phylogenetic analysis was performed based on the entire chloroplast genomes from foxtail millet, wheat, maize, rice and sorghum we reported here and five other Poaceae species available in NCBI (Fig. 4). The chloroplast genome of *Arabidopsis thaliana* (accession number AP000423.1) was used as an out-group. As shown in Fig. 4, Pooideae species, *Triticum aestivum* (Jinong6, Jinqiang8 and Lunxuan987) and *Branchypodium distachyon* (accession number NC_011032) formed a clade with Ehrhartoideae species, *Oryza sativa* (Jingeng818) and *Leersia tisserantii* (accession number NC_016677). Five Panicoideae species and one Chloridoideae species, *Cenchrus ciliaris* (accession number MH286942.1), formed another separate clade. In this clade, *Zea mays* (Zhengdan958) and *Sorghum bicolor* (SbJ200) were the earliest diverged species, followed by *Urochloa brizantha* (accession number NC_030067), then *Cenchrus americanus* (accession number NC_024171) and *Cenchrus ciliaris* were parallel to *Setaria italica* (Gu56A, Gu572A, Datong28 and Datong29lv).

## Discussion

Due to its highly conserved gene content and organization between species, cpDNA has been widely used to study genome-wide phylogenetics and ecology, transcriptomics, and complete plastid proteome characterization^21–23^. However, an effective method for cpDNA isolation from foxtail millet is currently lacking due to its complicated leaf structure, which makes it difficult to break cells to release chloroplasts^18^. In this study, we developed a new cpDNA extraction protocol that can be used for foxtail millet and several other crop species that have difficulty in cpDNA extraction (Fig. 1). Compared to previously reported methods^15–17^, the new protocol has been technically improved by the following steps: 1) using a surgical blade to cut leaves into tiny pieces instead of grinding tissue in a grinding machine; 2) extracting protoplasts instead of a homogenization process; 3) selecting appropriate centrifugation speeds and density gradients to separate chloroplasts from other DNA and cell components. These changes significantly increased chloroplast quality and solved several issues including broken chloroplasts from grinding the narrow and thin leaves of foxtail millet, contamination of cell debris in isolated chloroplasts due to the high content of minerals, and nuclear and mitochondrial DNA contamination. The new protocol protected the intactness of chloroplasts of foxtail millet, thus significantly improving chloroplast quality, which is critical for sequencing the chloroplast genome of foxtail millet and lays a solid foundation for further understanding the genetic basis of foxtail millet.

To determine the quality of isolated cpDNA, cpDNA of all five species with narrow and thin leaves was sequenced, and high proportions (4.22–12.35%) of reads were mapped onto their corresponding chloroplast genomes. The percentage of mapped reads was much higher than those (1% to 4%) from cpDNA isolated using other protocols^19^, indicating that the purity of the cpDNA isolated using the new protocol was much higher than that with existing protocols and is good enough for whole chloroplast genome assembly. We sequenced ten complete chloroplast genomes from five species (Fig. 2, Supplementary Figs S1–S4) using the new protocol and obtained high-quality sequence data, indicating that the newly developed cpDNA isolation protocol is suitable not only for foxtail millet but also other Poaceae species with narrow and thin leaves. Based on the chloroplast genomes we sequenced here and cpDNA sequences from five other Poaceae species available in NCBI, we constructed a phylogenetic tree using the maximum-likelihood method in MEGA 7.0 with 1000 replicates. The topology of this phylogenetic tree is consistent with a previous report^19^, indicating that the chloroplast genome sequences we reported here can be used for well-supported phylogenetic reconstruction, and these data will provide invaluable resources for future research.

We compared the chloroplast genome sequences of ten genotypes from five species to their reference genomes in NCBI (Fig. 2, Supplementary Figs S5–S8) and found that the maize cultivar Zhengdan958 had an identical chloroplast genome to its reference genome, the cultivar B73 (AY928077.1) (Supplementary Fig. S5). Moreover, the chloroplast genomes of the rice cultivar Jingeng818 and sorghum cultivar SbJ200 also showed high sequence similarity to their reference sequences (Supplementary Figs S6–S7), demonstrating the reliability of our sequence data. According to the maternal inheritance of cpDNA in plants, the completely matched chloroplast genomes of Zhengdan958 and B73 revealed that they have a common maternal parent. The pedigree analysis of Zhengdan958 reflected that Zhengdan958 was derived from the cross Zheng58 × Chang7-2, and Zheng58 was a variant from the inbred line Ye478 that was derived from U8112 × 5003^24,25^, demonstrating that Zhengdan958, Zheng58, Ye478 and U8112 derived from the same maternal parent. Thus, we speculated that Zhengdan958, Zheng58, Ye478, and U8112 may share a common maternal parent with B73.

Compared to the nuclear genome, a chloroplast genome is much smaller with a simpler structure and more conserved gene content and arrangement^5^; thus, the chloroplast genome is more useful for taxonomic studies. For instance, the chloroplast genes *matK* and *rbcL* have been developed as effective molecular markers for the identification of land plant species^26^. Non-coding regions in the chloroplast genome, such as the *trnL* intron, intergenic spacer *trnH-psbA* and *trnL*-*trnF*, have been widely used as chloroplast barcoding markers in plant systematics and phylogeography^27–31^. Comparative analysis of complete chloroplast genomes of four foxtail millet cultivars and three wheat cultivars identified several InDels in intergenic spacer regions and genic regions (Fig. 3, Supplementary Fig. S8). These InDels could be developed as DNA markers for the identification of accessions in germplasm collections and breeding in foxtail millet and wheat.

In some previous studies, independent deletions mediated by short direct-repeat sequences were successively found among the cpDNAs of three species of wheat, *Aegilops crassa*, *Aegilops squarrosa* and *Triticum aestivum*^32^, and four species of rice, *Oryza punctata*, *Oryza officinalis*, *Oryza australiensis* and *Oryza sativa*^33^. The variations in the deletions are genotype-specific and species-specific in rice and wheat respectively. In 1993, similar deletions were also found within the single species of *Oryza sativa*, suggesting that the occurrence of this deletion was comparatively easy between species and within a single species during evolution^34^. In this study, we sequenced the complete chloroplast genomes of four foxtail millet cultivars. Does this type of specific variation exist within these four cultivars or even more cultivars of foxtail millet? Where are the locations of these variations? Additionally, what are the sequences around them? These questions will be examined in our further studies and may provide a basis for cultivar classification if there are intraspecific variations of cpDNAs within foxtail millet.

## Materials and Methods

### Plant materials

Ten cultivars of five species were used as testing materials for protocol optimization and sequencing in this study, including a summer maize hybrid cultivar Zhengdan958, a Japonica rice cultivar Jingeng818, a sorghum cultivar SbJ200, a spring wheat cultivar Jinqiang8, two winter wheat cultivars Jinnong6 and Lunxuan987, and four foxtail millet cultivars Gu56A, Gu572A, Datong28 and Datong29lv. All plant materials were grown in the greenhouse of Tangshan Normal University, China. For each plant genotype, ~10 g of fresh leaves were harvested at the 6th to 8th leaf stage for chloroplast extraction.

### Reagent preparation

Enzyme solution containing 0.5% cellulase R10 (w/v), 0.3% macerozyme R10 (w/v), 0.4 M mannitol, 20 mM KCl, and 20 mM MES (pH 5.7) was prepared by warming at 55 °C for 10 min, cooling to room temperature (25 °C), and then adding 10 mM CaCl_2_ and 0.1% BSA (w/v). Buffer 1 contained 0.9% NaCl (w/v), 1.4% CaCl_2_ (w/v), 0.04% KCl (w/v), 0.1% glucose (w/v), and 1 mM MES (pH 5.7). Buffer 2 contained 0.3 M sorbitol, 1 mM MgCl_2_·6H_2_O, 2 mM EDTA·2Na, 50 mM HEPES, and 0.04% β-mercaptoethanol (w/v), and Buffer 3 consisted of 0.3 M sorbitol, 1 mM MgCl_2_·6H_2_O, 2 mM EDTA·2Na, and 50 mM HEPES.

### Chloroplast DNA isolation

#### Protoplast isolation

Leaves were cut into 0.5–1-mm strips by a sharp razor blade without crushing tissue at the cutting site and completely submerged into the enzyme solution (10 g of leaves in 50–150 ml) for digestion at room temperature in the dark for at least 3 h without shaking. The enzyme solution was diluted with an equal volume of Buffer 1 and filtered through a copper mesh (cell strainer, 200 meshes) to remove undigested leaf tissues. The filtered solution was centrifuged at 200 g afterwards to pellet the protoplasts in a 50-ml round-bottomed tube for 10 min at 4 °C.

#### Crude chloroplast extraction

The protoplasts were re-suspended in 50 ml of Buffer 2, kept on ice and inverted once every 5 min. After 15 min, the protoplasts were centrifuged at 500 g for 10 min at 4 °C, and the supernatant was harvested gently. The supernatant was then centrifuged at 3000 g for 15 min at 4 °C, and the resulting crude chloroplast precipitate was collected.

#### Chloroplast purification and cpDNA isolation

The crude chloroplasts were re-suspended with Buffer 3 (500–1000 µl), layered onto a Percoll gradient (10–50% for foxtail millet, 25–45% for wheat, 10–45% for maize, 15–50% for rice and 15–45% for sorghum) made with the same buffer, and centrifuged at 3000 g for 30 min at 4 °C. The resulting chloroplasts between the two gradients were collected, re-suspended in 1 ml of Buffer 3, and centrifuged at 12000 rpm for 5 min at 4 °C. The supernatant was removed, and the last step was repeated once. The high-purity chloroplast pellets were then collected, frozen with liquid nitrogen and stored at −80 °C for cpDNA isolation.

cpDNA was extracted from the intact chloroplasts using the DNAquick Plant System DP321 (Tiangen Biotech, Beijing, China) following the manufacturer’s instructions.

### Whole chloroplast genome sequencing, assembly and annotation

The cpDNAs were sequenced in an Illumina HiSeq Sequencer at Beijing Ori-Gene Science and Technology (China). Low-quality reads, reads with adaptor sequences and duplicated reads were removed, and the remaining high-quality data were used for assembly. The clean reads were first combined into contigs using SOAPdenovo^35^, Then, the assembled contigs were aligned to the reference genome to detect assembly errors using BLAT^36^. The gaps between contigs were further bridged using the GapCloser package to refine the assembly. Finally, the physical map was constructed using OrganellarGenomeDRAW^37^ by anchoring the scaffolds onto chloroplast genome sequences.

Sequenced genomes were annotated using CpGAVAS^38^. This program identified both protein-coding genes and rRNA genes by performing Blastx, Blastn, protein2genome and est2genome^39^ searches against a custom database of published plastid genomes. tRNAs were identified by tRNAscan^40^ and ARAGORN^41^. Inverted repeat regions (IRs) were identified using vmatch^42^.

### Sequence alignment and phylogenetic analysis

Chloroplast genome sequences were aligned using VISualization Tool for Alignments (mVISTA) (http://genome.lbl.gov/vista/index.shtml)^20^. Phylogenetic analysis was performed using the maximum likelihood method, as implemented in the MEGA 7.0 program with 1000 bootstrap replicates^43^.

## Supplementary information

Supplementary Information

Dataset 1

## Data Availability

The chloroplast genome sequences we reported in this study and corresponding annotations have been deposited to GenBank under the following accession numbers: 56 A (MK348603), 572 A (MK348609), Datong28 (MK348605), Datong29lv (MK348604), Jinnong6 (MK348601), Jinqiang8 (MK348611), Lunxuan987 (MK348610), Zhengdan958 (MK348606), Jingeng818 (MK348618), SbJ200 (MK348612).
