# Supplementary material for: A New Chloroplast DNA Extraction Protocol Significantly Improves the Chloroplast Genome Sequence Quality of Foxtail Millet (Setaria italica (L.) P. Beauv.)
Source: Sci Rep. 2019 Nov 7;9:16227. doi: 10.1038/s41598-019-52786-2 (PMC6838068; doi:10.1038/s41598-019-52786-2)
Supplement: Supplementary file 1 — Supplementary Information [file 41598_2019_52786_MOESM1_ESM.pdf]

Supplementary data

Article title: A New Chloroplast DNA Extraction Protocol Significantly Improves the Chloroplast Genome Sequence Quality of Foxtail Millet (*Setaria italica* (L.) P. Beauv.)

Authors: Dan Liu, Yanjiao Cui, Suying Li, Guihua Bai, Qiang Li, Zilong Zhao, Dan Liang, Conglei Wang, Jianhe Wang, Xiaowei Shi, Chao Chen, Gang Feng, and Zhengli Liu

The following Supporting Information is available for this article:

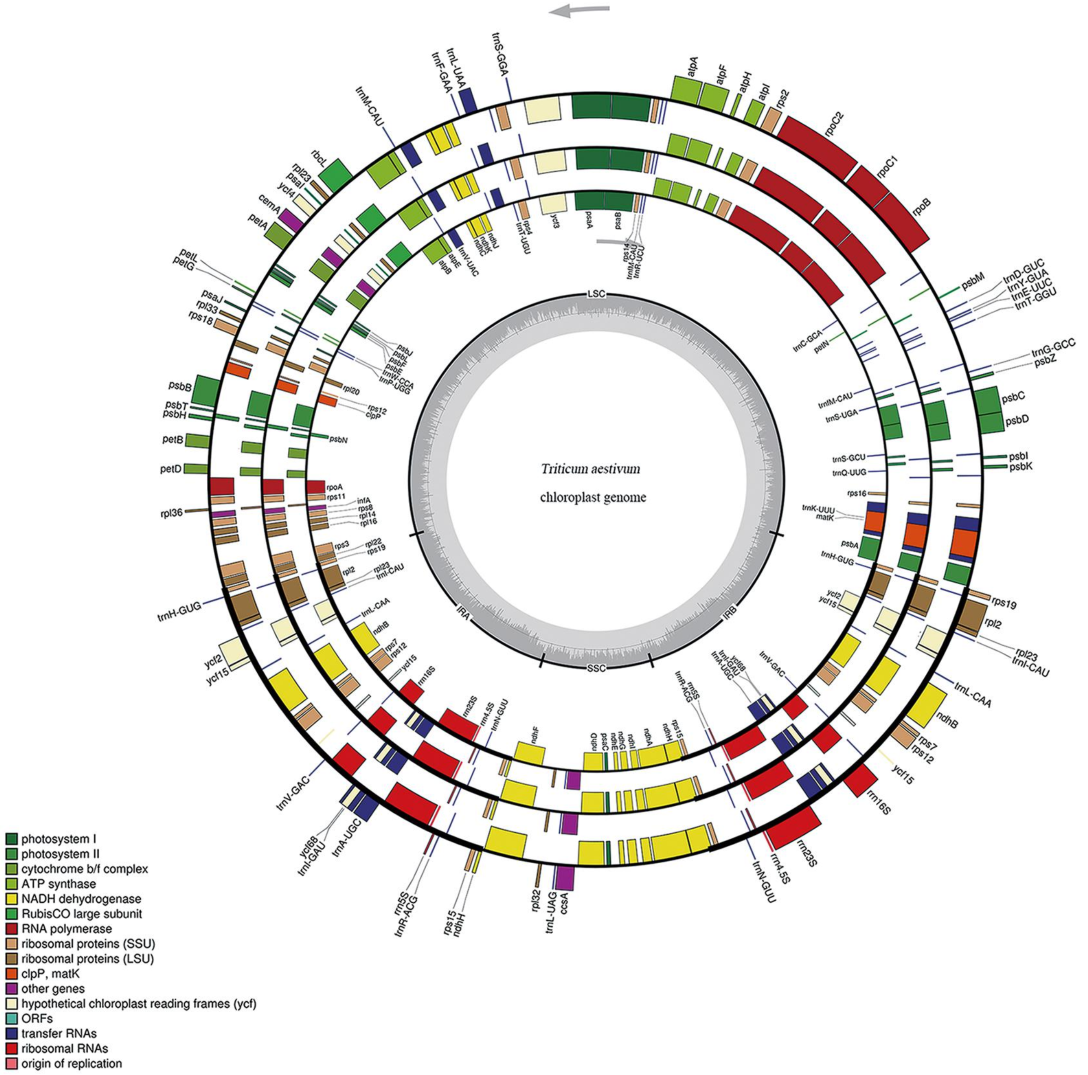

Figure S1. Genome structure and mapping of genes in the wheat chloroplast genome. The wheat cultivars from inside to outside are Jinnong6, Lunxuan987 and Jinqiang8, respectively. The thick lines indicate the extent of the IRA and IRB, which separate the genome into the SSC and LSC regions. Genes on the outside of the map are transcribed in the counterclockwise direction, and genes on the inside of the map are transcribed in the clockwise direction. Genes are colored according to their functions as shown in the legend.

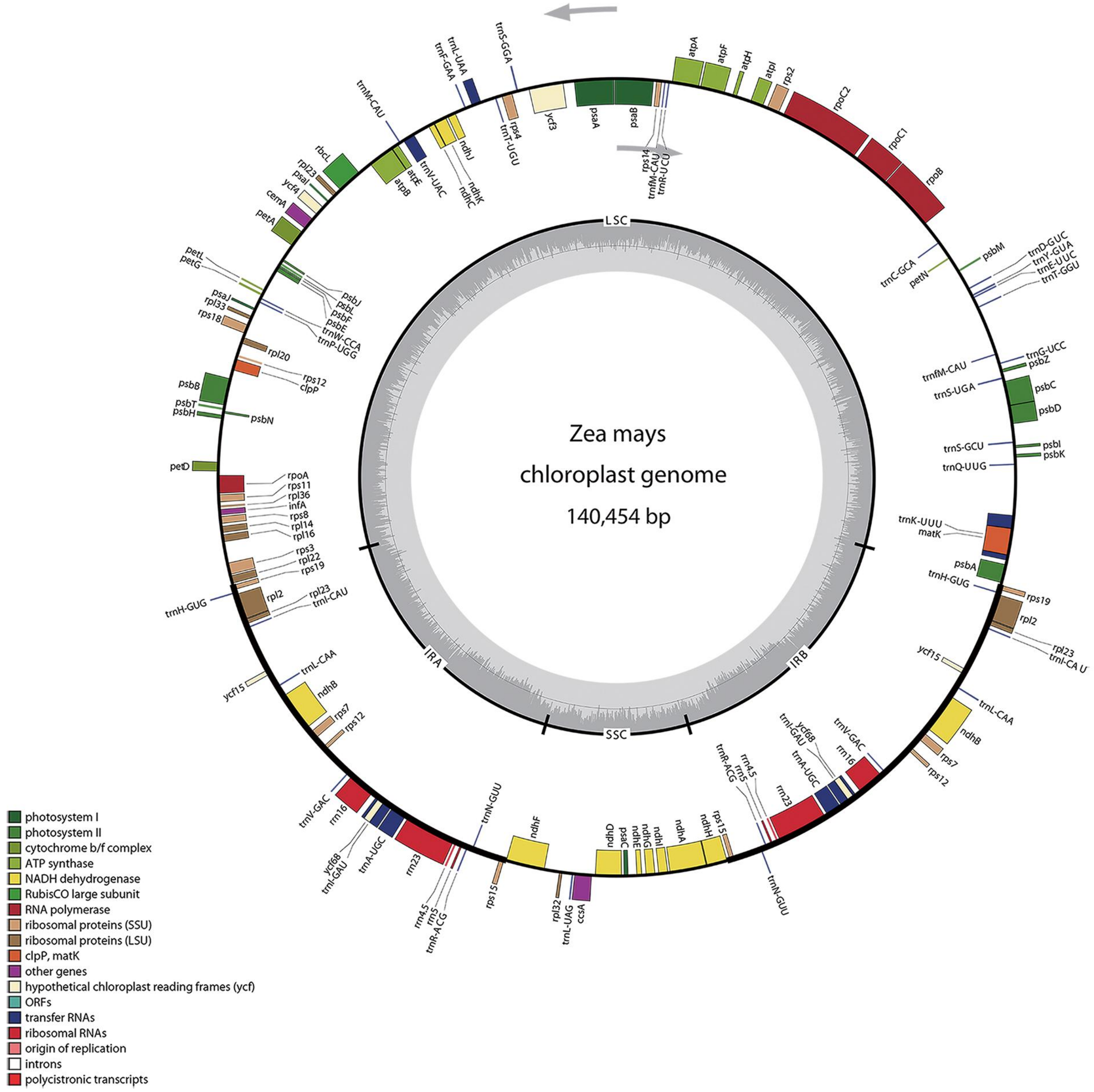

Figure S2 Genome structure and mapping of genes in the maize chloroplast genome. The thick lines indicate the extent of the IRA and IRB, which separate the genome into the SSC and LSC regions. Genes on the outside of the map are transcribed in the counterclockwise direction, and genes on the inside of the map are transcribed in the clockwise direction. Genes are colored according to their functions as shown in the legend.

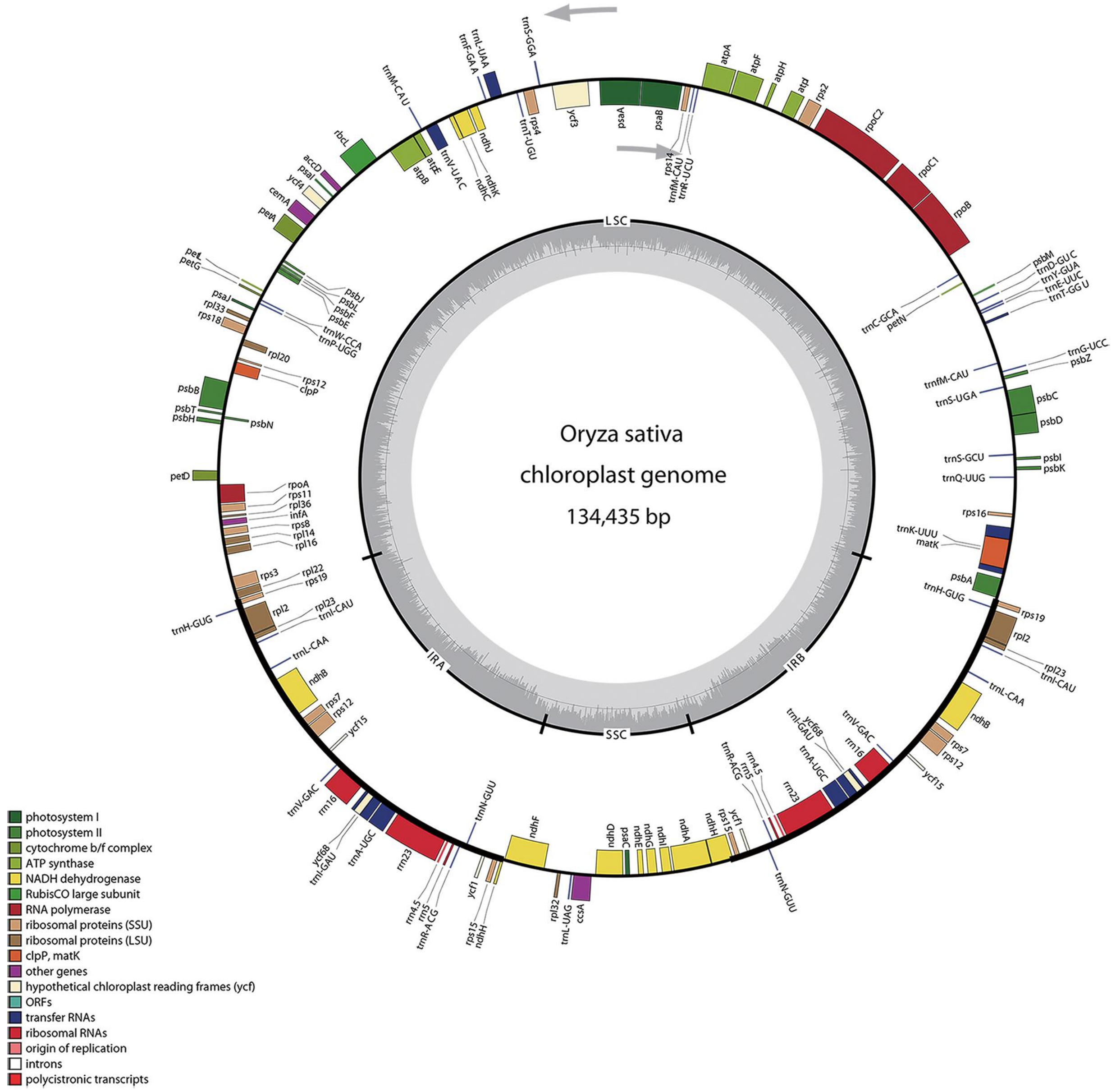

Figure S3 Genome structure and mapping of genes in the rice chloroplast genome. The thick lines indicate the extent of the IRA and IRB, which separate the genome into the SSC and LSC regions. Genes on the outside of the map are transcribed in the counterclockwise direction, and genes on the inside of the map are transcribed in the clockwise direction. Genes are colored according to their functions as shown in the legend.

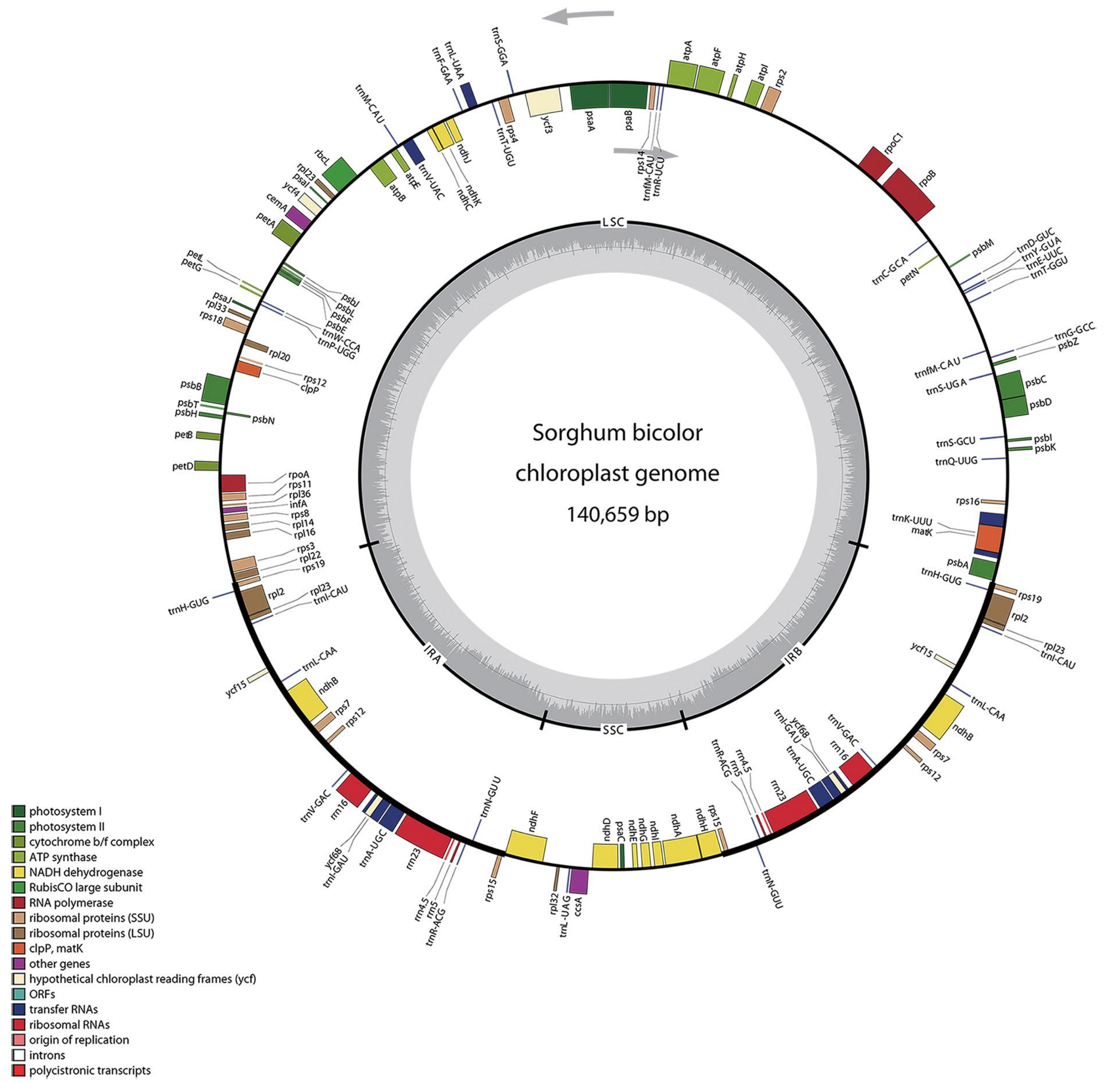

Figure S4 Genome structure and mapping of genes in the sorghum chloroplast genome. The thick lines indicate the extent of the IRA and IRB, which separate the genome into the SSC and LSC regions. Genes on the outside of the map are transcribed in the counterclockwise direction, and genes on the inside of the map are transcribed in the clockwise direction. Genes are colored according to their functions as shown in the legend.

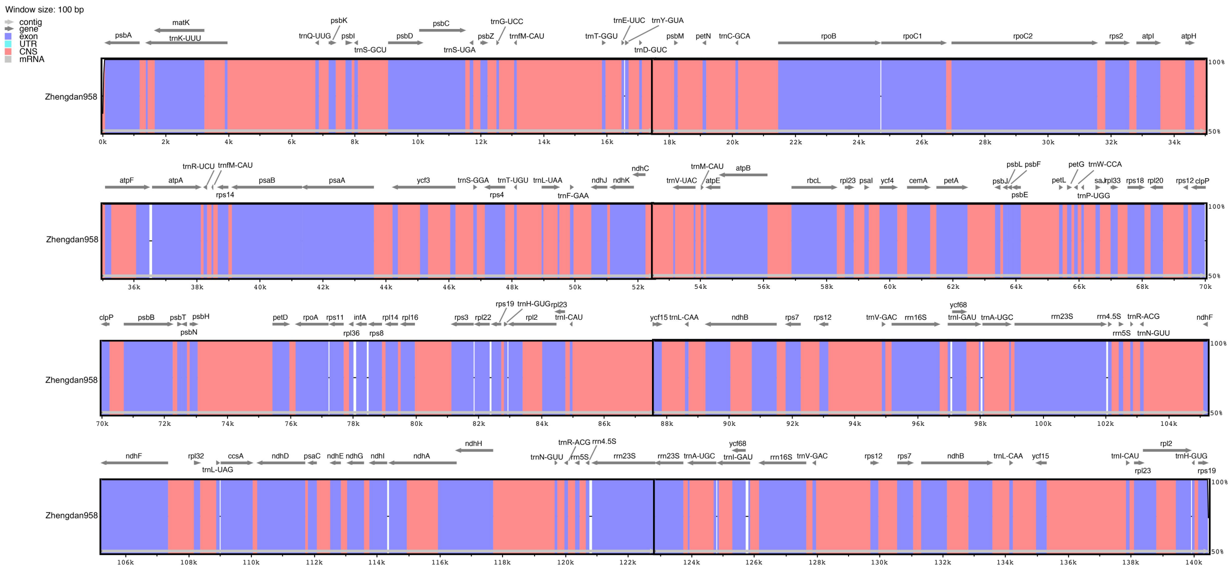

Figure S5 Alignment and percentage identity of complete chloroplast genome sequences of maize cultivars Zhengdan958 and B73 (AY928077.1) using mVISTA. Exons are shaded in dark blue, and the conserved non-coding sequences (CNS) are shaded in pink. Arrows indicate positions of annotated genes in reference sequences.

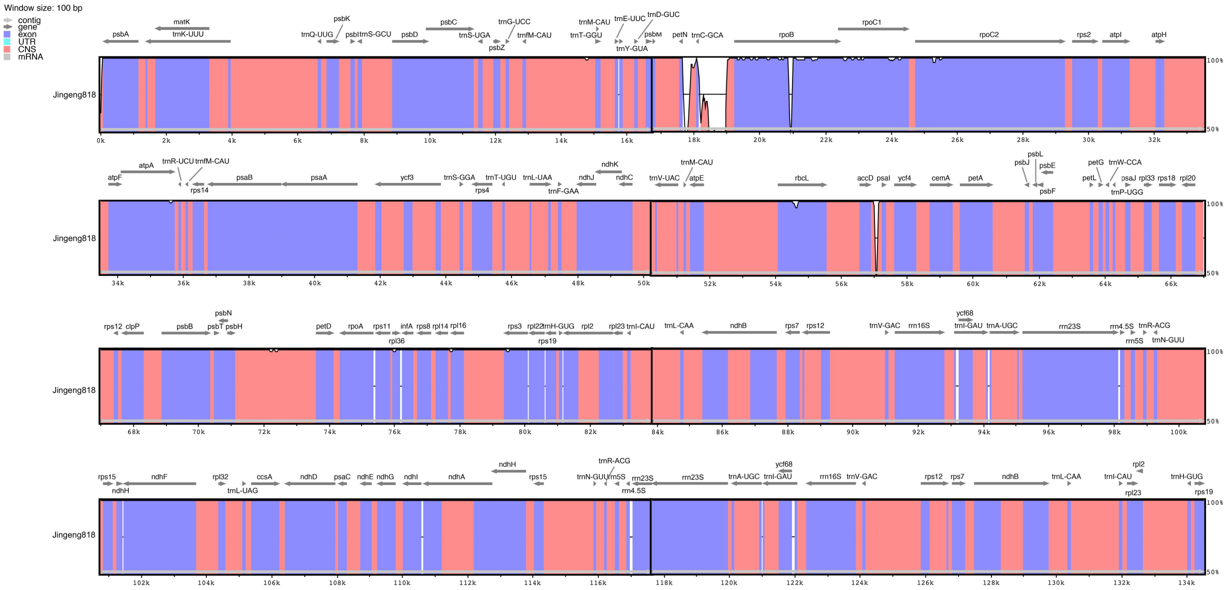

Figure S6 Alignment and percentage identity of complete chloroplast genome sequences of rice cultivars Jingeng818 and Nipponbare (GU592207.1) using mVISTA. Exons are shaded in dark blue, and the conserved non-coding sequences (CNS) are shaded in pink. Arrows indicate positions of annotated genes in reference sequences.

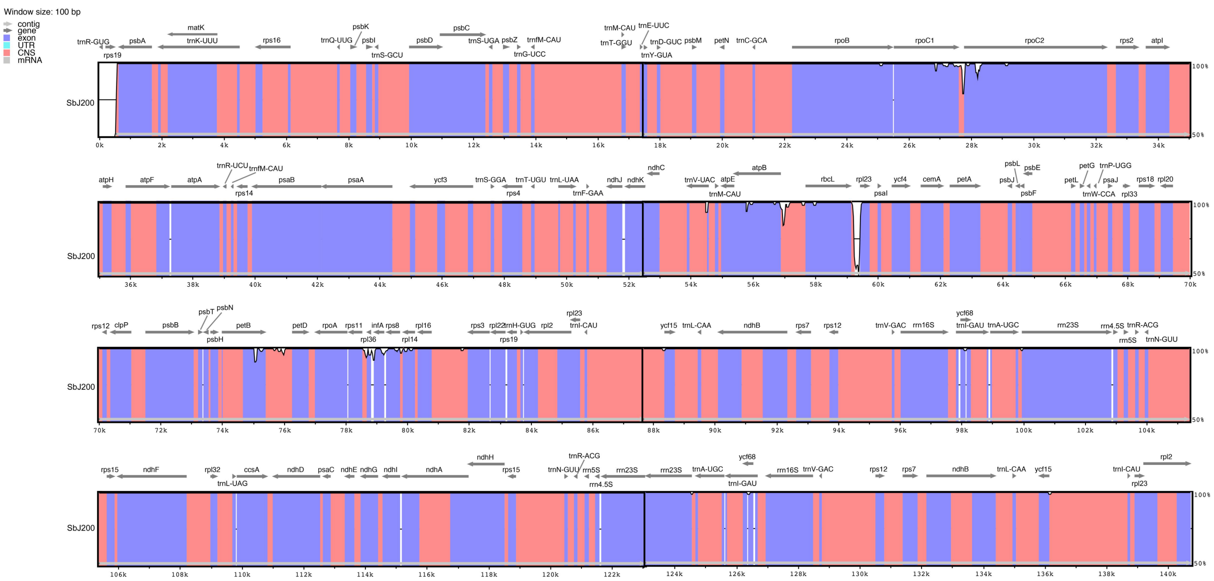

Figure S7 Alignment and percentage identity of complete chloroplast genome sequences of sorghum cultivars SbJ200 and BTx623 (EF115542.1) using mVISTA. Exons are shaded in dark blue, and the conserved non-coding sequences (CNS) are shaded in pink. Arrows indicate positions of annotated genes in reference sequences.

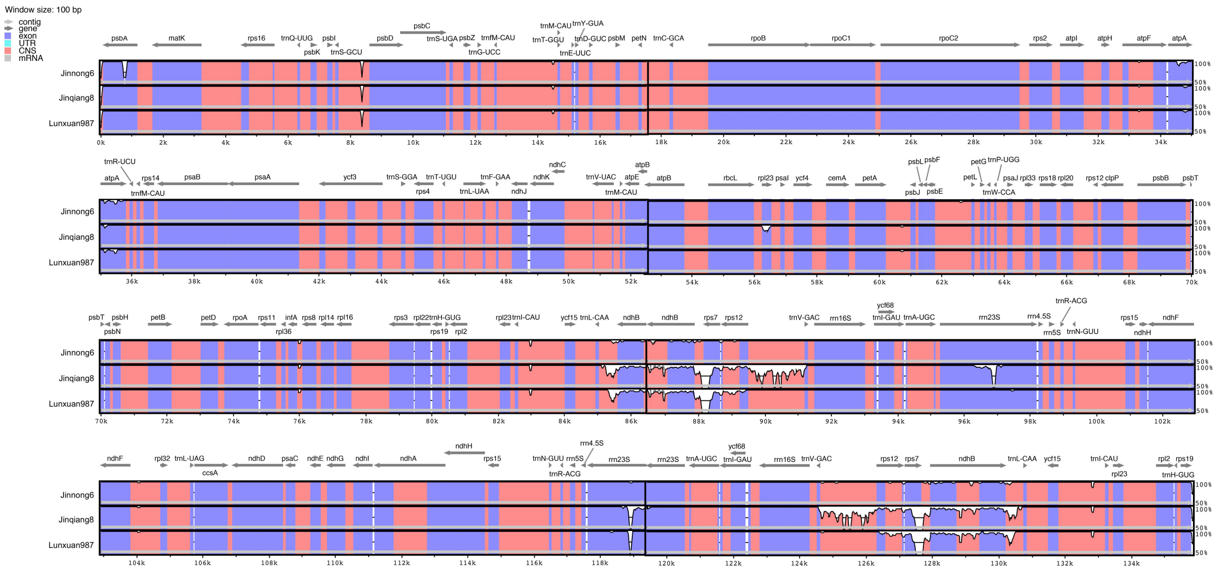

Figure S8 Alignment and percentage identity of complete chloroplast genome sequences of four wheat cultivars using mVISTA. Measures of similarity are relative to reference chloroplast genome KJ614396.1. Exons are shaded in dark blue, and the conserved non-coding sequences (CNS) are shaded in pink. Arrows indicate positions of annotated genes in reference sequences.

Table S1 Statistics on the basic features of the chloroplast genomes of three wheat cultivars sequenced in our study.

	Jinqiang8	Jinnong6	Lunxuan987
Length (bp)	135574	136482	135437
GC content (%)	38.19	38.35	38.26
AT content (%)	61.81	61.65	61.74
LSC length (bp)	79990	80594	79993
SSC length (bp)	12790	12790	12790
IR length (bp)	21397	21549	21327
Gene number	136	129	134
Gene number in IR regions	40	32	38
Protein-coding gene number	96	89	94
Protein-coding gene (%)	70.59	68.99	70.15
rRNA gene number	8	8	8
rRNA (%)	5.88	6.2	5.97
tRNA gene number	32	32	32
tRNA (%)	23.53	24.81	23.88

Table S2 Statistics on the basic features of the chloroplast genomes of maize, rice and sorghum cultivars sequenced in our study.

	Zhengdan958	Jingeng818	SbJ200
Length (bp)	140454	134435	140659
GC content (%)	38.44	39.08	38.51
AT content (%)	61.56	60.92	61.49
LSC length (bp)	82382	80488	82592
SSC length (bp)	12540	12343	12503
IR length (bp)	22766	20802	22782
Gene number	132	136	133
Gene number in IR regions	40	42	40
Pseudogene number	2	3	2
Pseudogene (%)	1.52	2.21	1.5
Protein-coding gene number	84	87	85
Protein-coding gene (%)	63.64	63.97	63.91
rRNA gene number	8	8	8
rRNA (%)	6.06	5.88	6.02
tRNA gene number	38	38	38
tRNA (%)	28.79	27.94	28.57
